# Impact of COVID-19 vaccination on radiation-induced lung injury in breast cancer patients: a retrospective cohort study

**DOI:** 10.1007/s12672-026-04899-0

**Published:** 2026-03-26

**Authors:** Tamás Ungvári, Zsanett Ungváriné Topcsiov, András Kedves, Döme Szabó, Balázs Kiss, Zsófia Dankovics, Judit Olajos, Károly Tőkési

**Affiliations:** 1 Department of Oncoradiology, Markusovszky University Teaching Hospital, Hungary 9700 Szombathely Markusovszky str. 5, Szombathely, Hungary; 2 Department of Centralized Laboratory, Markusovszky University Teaching Hospital, Szombathely, Hungary; 3 Department of Oncoradiology Nyíregyháza, Jósa András University Teaching Hospital, Nyíregyháza, Hungary; 4 Institute of Physical Education and Sport Science, University of Nyíregyháza, Nyíregyháza, Hungary; 5 HUN-REN Institute for Nuclear Research (Atomki), 4026 Debrecen Bem tér 18/c, Debrecen, Hungary

**Keywords:** Radiation-induced lung injury, COVID-19 vaccination, Radiotherapy, Breast cancer

## Abstract

**Background:**

The safety of COVID-19 vaccination during radiotherapy remains a concern among oncologists. This study investigates whether vaccination increases the risk of radiation-induced lung injury (RILI) in breast cancer patients.

**Methods:**

We retrospectively analyzed 157 breast cancer patients treated with radiotherapy between April 2021 and October 2022. Lung injury was assessed through post-treatment imaging and correlated with COVID-19 vaccination status.

**Results:**

Of 157 patients, 70 (44%) developed radiologic signs of radiation induced lung injury. Most patients (85%) were vaccinated. Statistical analysis revealed no significant association between vaccination status and injury risk (Chi-square *p* = 0.3569; logistic regression *p* = 0.646, OR = 1.063, 95% CI: 0.821–1.383). A non-significant trend toward reduced injury was noted in the two-dose subgroup.

**Conclusion:**

The findings of this study indicate that the administration of the COVID-19 vaccine prior to the commencement of radiation therapy is not associated with an increased likelihood of pulmonary toxicity. Although a slight protective effect was observed with two vaccine doses, it was not statistically significant. The retrospective nature of the study and the limited sample size necessitate the execution of larger, prospective, long-term follow-up studies to elucidate the underlying immunological mechanisms.

## Background

While the mortality rate of breast cancer has declined due to advances in early detection and treatment, it remains the most prevalent cancer among women. The five-year survival rate exceeds 90%, underscoring the importance of minimizing long-term side effects that impact quality of life [1].

Radiotherapy is a cornerstone of breast cancer treatment following surgery, as it substantially reduces the risk of recurrence [2]. However, it may lead to radiation-induced lung injury (RILI), which typically progresses in two distinct phases: acute radiation pneumonitis and chronic radiation-induced lung fibrosis [3, 4, 5].

Pulmonary fibrosis is an irreversible and progressive condition that compromises respiratory function and diminishes long-term survival. Its pathogenesis involves several mechanisms, including the generation of reactive oxygen species during irradiation, immune-mediated processes, and elevated levels of pro-fibrotic cytokines [6, 7, 8]. Patient-specific factors, such as smoking history, pre-existing lung disease, and concomitant medications, may further modulate susceptibility to fibrosis [9, 10, 11]. Moreover, technical parameters such as total radiation dose, field size, and the volume of lung tissue exposed are known to affect the incidence of radiation-induced lung fibrosis [12, 13].

The COVID-19 pandemic has brought increased attention to pulmonary pathologies and their interaction with the immune system. Although COVID-19 infection has been linked to lung fibrosis, the impact of vaccination in this context remains unclear [14, 15, 16]. Some studies suggest that vaccination may mitigate pulmonary complications by reducing inflammation, while others raise concerns that immune activation might contribute to fibrotic processes [17, 18, 19]. Additionally, systemic inflammation triggered by severe COVID-19 may aggravate pre-existing lung damage and potentially alter the effects of radiation therapy in cancer patients [20].

Given the widespread uptake of COVID-19 vaccines and their known immune effects, their influence on radiation-induced lung injury is a matter of clinical relevance. Breast cancer patients offer a suitable population for this investigation due to the frequent involvement of lung tissue during radiotherapy. Furthermore, the lungs—owing to their high radiosensitivity and immunological reactivity—provide an ideal model for studying the intersection of vaccination and radiation-induced toxicity [21, 22, 23].

Emerging evidence suggests that systemic immune activation, modulated by vaccination, may influence fibrotic responses. While some data point to a potential protective role through inflammation reduction, others hypothesize that vaccine-induced immune stimulation could exacerbate fibrotic pathways. Clarifying this relationship is essential for optimizing radiotherapy protocols and follow-up strategies in oncology.

Therefore, the aim of this study was to evaluate whether COVID-19 vaccination status is associated with the development of radiation-induced lung injury in breast cancer patients. By analyzing post-radiotherapy imaging data in relation to vaccination status, we sought to determine whether immunization plays a measurable role in modifying pulmonary toxicity outcomes.

## Methods

### Population

This retrospective study included 157 breast cancer patients with stage I–III invasive adenocarcinoma or carcinoma in situ who underwent radiotherapy [24] between April 2021 and October 2022. Only patients with available post-treatment chest CT or X-ray imaging were included.

### Data collection

The protocol specified that the first imaging scan would be conducted three months after radiotherapy. Depending on the patient’s condition and tumor stage, the second CT or chest X-ray was scheduled for either six or nine months after the completion of radiotherapy. It is important to note that the actual dates may vary based on the patient’s availability for the scan. Patients who requested COVID-19 vaccination received it either before surgery or, at the latest, prior to the initiation of radiotherapy. The treating physician verified vaccination status at baseline in the hospital records. The exact timing of additional booster doses in relation to radiotherapy could not be consistently determined.

Information on systemic therapies (chemotherapy, hormone therapy) and pulmonary comorbidities (e.g., COPD, interstitial lung disease, smoking status) was not consistently available in the institutional registry and therefore could not be included in this analysis.

### Data definitions and statistics

Radiological images from chest CT (78 patients) scans and X-rays (79) were evaluated for signs of lung injury following breast irradiation. Vaccination status was originally recorded in seven categories (0–6 doses). However, the subgroup of patients who received a single dose and suffered from injury consisted of only three patients, similar to the group of four patients without injury. The groups that received 4, 5, or 6 doses were also very small. To avoid rare cells and unstable estimates, and to maintain interpretability, these categories were combined into broader groups: 0–1, 2, 3, and ≥ 4 doses.

Radiological images from chest CT scans and X-rays were retrospectively evaluated for radiological signs consistent with RILI, with particular focus on pulmonary fibrosis-like changes (e.g., ground-glass opacities, reticular patterns, fibrotic scarring). It is important to note that our study assessed radiological features suggestive of RILI and fibrosis, but histopathological confirmation of RILI was not performed. By including vaccination status as an independent variable, we aimed to determine if a higher number of vaccine doses was associated with an increased or decreased risk of lung injury. Radiologists who assessed the imaging had no information about the patients’ COVID-19 vaccination status or radiotherapy treatment plans. Radiation-induced changes were differentiated from COVID-related pneumonia or other lung abnormalities based on their typical localization within the irradiated lung volume and the sharp demarcation along treatment fields. This contrasts with the diffuse ground-glass patterns commonly observed in viral pneumonia.

Statistical analysis was generated using Python 3.10 and Microsoft Excel 2013. Data processing and manipulation were performed using the pandas and NumPy libraries. The 3D rendering and plotting were achieved with Matplotlib (utilizing the mpl_toolkits.mplot3d submodule), seaborn, and the surface interpolation was conducted using functions from the SciPy library (scipy.interpolate). We applied the Chi-squared test to analyze the data. Additionally, we conducted a logistic regression analysis to assess whether COVID-19 vaccination influenced the likelihood of developing radiation-induced lung injury.

### Radiation technique and dosimetry

All patients underwent breast irradiation using either a 3D conformal or Intensity Modulated Radiotherapy technique, with CT-based planning utilizing mixed energies of 6 MV and 10 MV or 6 MV and 18 MV, respectively. The irradiation plan included tangential fields and additional beams to optimize target volume coverage while minimizing radiation exposure to the organs at risk, specifically the heart, lungs, and contralateral breast.

It is important to note that hypofractionated total breast irradiation can serve as an effective alternative to standard radiotherapy for women who have undergone breast-conserving surgery for invasive breast cancer, provided they have clear surgical margins and negative axillary nodes [25, 26].

Defining the target volume and the volume of the organs at risk is a crucial aspect of radiotherapy. Accurately identifying these volumes on the planning CT can be challenging. In our study, we adhered to the recommendations of the European Society for Radiotherapy and Oncology [27].

For the whole breast, a dose of 40.05 Gy [28, 29, 30, 31] was prescribed in 15 fractions (140 cases), while 50 Gy [28, 31] was given in 25 fractions (17 cases), based on pathological risk factors.

The supraclavicular lymphatic area and boost dosimetry for the tumor bed were excluded since tangential field placement was utilized, which did not significantly contribute to lung exposure. Lung contours were generated using the Siemens SOMATOM Go.Sim (Siemens, Erlangen, Germany) CT simulator software (Syngo.via). This automatic segmentation minimizes inter-observer variability in contouring organs at risk, resulting in more consistent data for normal tissue complication probability models. The delineated volumes allowed for accurate calculation of the projected radiation dose across different structures. The resulting dose-volume histogram illustrated the relationship between the dose (in Gy) and the volume percentage of a given structure [32, 33]. For each patient, the dose-response relationship could be calculated, independent of the radiotherapy technique or treatment volume. Lung volumes and mean lung doses [34, 35, 36] were derived from our treatment planning systems (Pinnacle version 16.2.1; Philips Radiation Oncology, Fitchburg, WI and Eclipse version 16.1; Varian Medical System, Palo Alto, CA).

## Results

Between 2021 and 2022, 157 patients were included in the study at the Markusovszky University Teaching Hospital in Szombathely. The average age of the patients at the time of treatment was 63 years. Among them, 70 patients (44%) developed a radiation-associated lung injury, while 87 patients (56%) had negative imaging findings. All cases were graded as 1 according to the Common Terminology Criteria for Adverse Events version 5. No medical intervention was required, as only radiological lesions were visible.

A total of 134 patients (85%) received at least one dose of a COVID-19 vaccine. The median follow-up was 13.5 months, with a range of 3 to 24 months, after the completion of radiotherapy. The patients’ data is summarized in Table 1.

When analyzing laterality, 53% of patients received left-sided irradiation and 47% received right-sided irradiation (OR = 0.6). There was a lower probability of developing lung injury with left-sided irradiation, possibly due to reduced doses reaching the lungs from the Deep inspiration breath-hold (DIBH) treatment or protective measures applied to the heart (OR = 0.53). Of the 31 patients treated with the DIBH technique, 10 developed RILI, while 21 experienced no side effects.

Regarding mastectomy, 32% of patients had undergone the procedure, while 68% had not (OR = 1.179). The likelihood of experiencing side effects was slightly higher in patients who had undergone a mastectomy compared to those who had not.

Patients who had undergone mastectomy exhibited a slightly higher rate of RILI (48%) compared to those without (43%), though this difference was not statistically significant (*p* = 0.6774). Mastectomy can influence radiation planning, field size, and dose distribution, which may in turn affect lung dose metrics.

We note, however, that the lung dose-volume parameters did not differ significantly between patients with and without RILI. The mean ipsilateral lung V17 Gy (defined as the percentage of the ipsilateral lung volume encompassed by the 17 Gy isodose) was 19.6% (± 2.5) in patients with RILI, compared to 18.8% (± 3.0) in those without fibrosis. Similarly, the mean lung dose was 8.6 Gy (± 1.3) for patients with fibrosis versus 8.2 Gy (± 1.2) for those without.

**Table 1:** Descriptive statistics. the summary of the patient data

	Without RILI number of patients	With RILI number of patients
Median age	63	64,5
Min age	34	37
Max age	89	85
Above 80	9	7
71–80	19	15
61–70	26	19
51–60	18	20
41–50	13	8
30–40	2	1
Laterally of the breast		
Left side	46	28
Rright side	41	42
Dosage of Irradiation		
15 × 2,67 Gy	76	64
25 × 2 Gy	11	6
Mastectomy		
Yes	26	24
No	61	46
Lung Volume cm^3^		
Above 80	1469,425	990,44
71–80	1371,305263	1420,72
61–70	1672,209375	1505,384211
51–60	2015,261111	1771,25
41–50	1781,661538	1859,675
30–40	1419,7	2658,9
Vaccination	Number of patient	Number of patient
6	1	0
5	0	1
4	4	6
3	44	39
2	22	9
1	3	4
0	13	11
Sum vaccinated patients	87	70

**Fig. 1:**
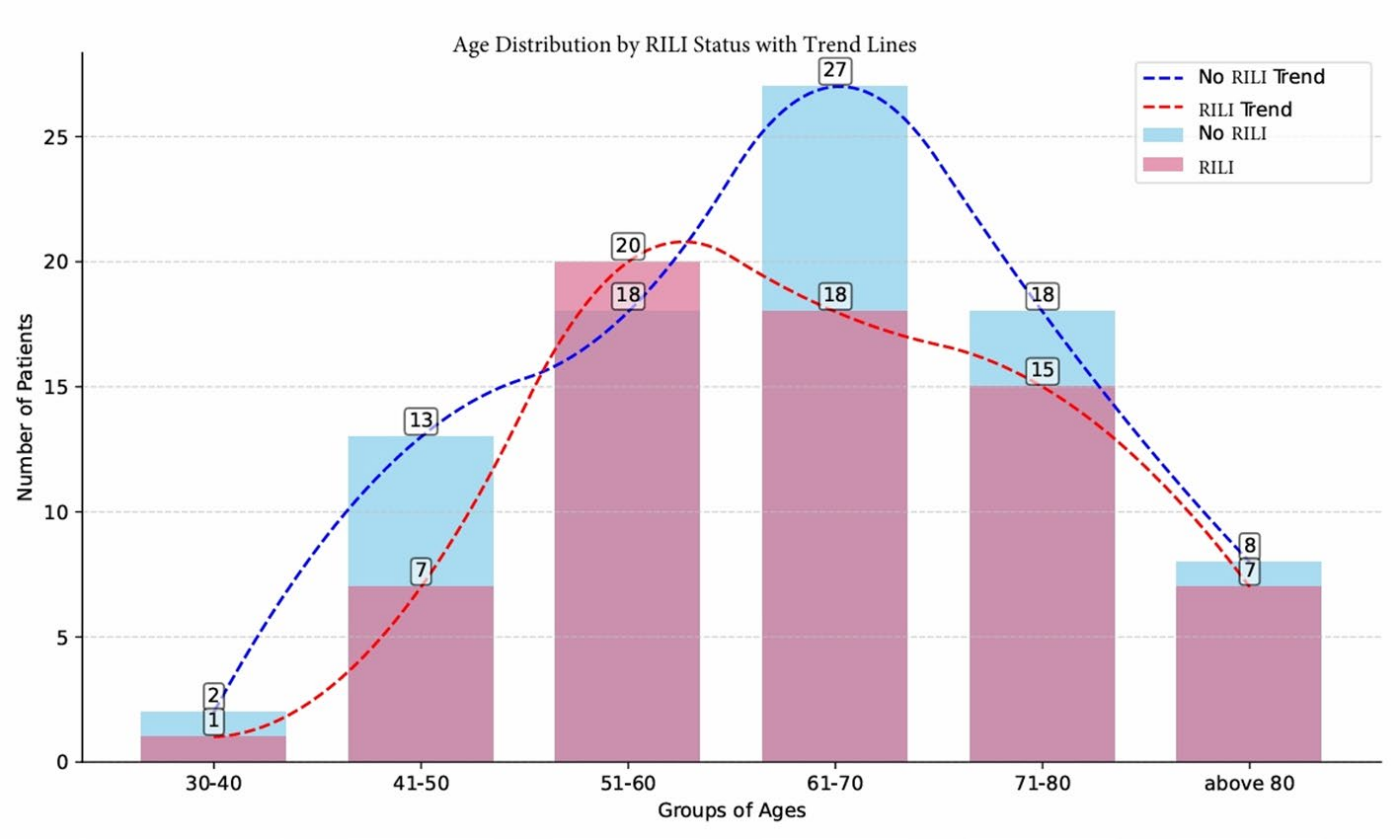
Age distribution of patients stratified by RILI status with superimposed trend lines. This histogram presents the distribution of patients across different age groups (30–40, 41–50, 51–60, 61–70, 71–80, and above 80 years), segmented by the presence RILI (fibrosis) or absence (Non-Fibrosis) The stacked bars illustrate the number of patients within each age stratum for both groups

The age distribution in patients with and without RILI is shown in Fig. 1. The median age was 64.5 years in the RILI group and 63 years in the non-injury group.

The coronavirus disease 2019 (COVID-19) pandemic has had a major impact on both diagnosis and treatment. Last but not least, we aimed to assess whether COVID-19 had an impact on the side effects of breast irradiation. We found that the majority of patients received the recommended 3 vaccinations.

The Chi-squared test revealed no statistically significant association between vaccination status and post-radiotherapy pulmonary RILI (*p* = 0.3569). While patients who received two vaccine doses exhibited a lower RILI rate (22 cases compared to 9), this difference was not statistically significant.

**Fig. 2:**
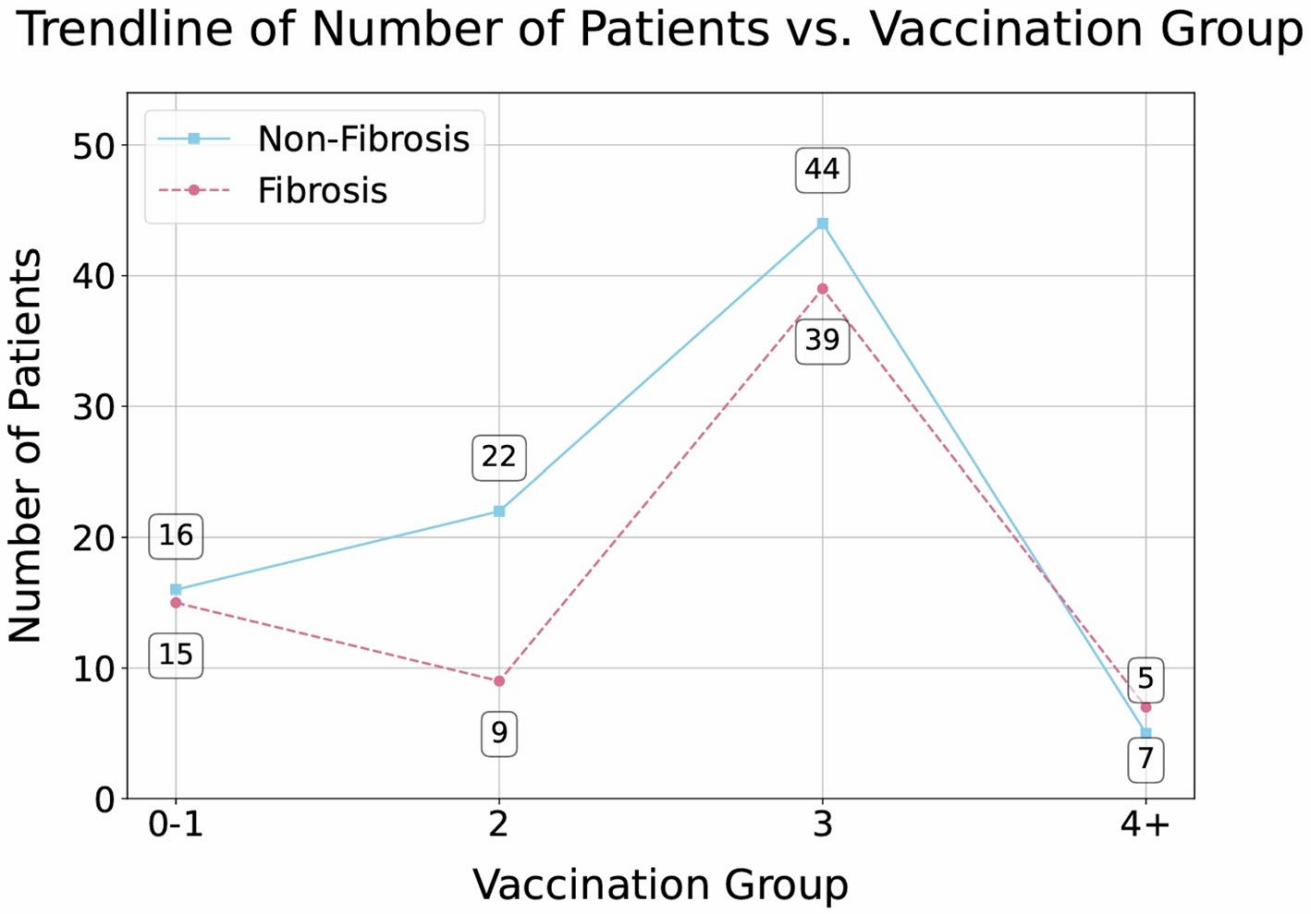
Distribution of patients across vaccination groups stratified by RILI Status (Fibrosis vs. Non-Fibrosis). This trendline illustrates the number of patients within each vaccination group (0–1, 2, 3, and 4 + doses) and further delineates these counts based on the presence (Fibrosis) or absence (Non-Fibrosis)

The proportion of adverse events was nearly identical among those vaccinated three times (44 vs. 39) (see Fig. 2).

Logistic regression analysis was conducted to evaluate whether the number of vaccine doses could predict the likelihood of developing RILI. The model included vaccination status as the independent variable and the occurrence of RILI as the dependent variable. The analysis showed that vaccination status did not significantly predict RILI development (*p* = 0.646, OR = 1.063, 95% CI: 0.821–1.383). The model’s pseudo R² value was 0.00098, indicating minimal explanatory power. Although patients who received two doses exhibited a lower incidence of RILI (22 vs. 9 cases), this trend did not reach statistical significance. Overall, the results suggest no clear protective or adverse effect of vaccination on radiation-induced lung injury.

Among patients who underwent mastectomy, 48.00% have RILI. Among patients who did NOT undergo mastectomy, 42.99% have RILI. According to the Chi-square, the P-value (0.6774) is greater than or equal to the significance level (0.05). Therefore, there is no statistically significant association between mastectomy status and RILI.

## Discussion

The observed distribution of COVID-19 vaccination doses reflects the structured, age-prioritized vaccination strategies implemented during the pandemic, which were largely consistent with international recommendations. Similar approaches were adopted both nationally and internationally, including in Hungary and other countries such as Mexico [37].

We also evaluated whether the laterality of irradiation—left or right side—had an impact on RILI rates. Our findings showed a slightly reduced RILI incidence in left-sided treatments (OR = 0.53), possibly reflecting anatomical and planning-related factors that result in lower ipsilateral lung exposure. While the exact mechanism remains speculative, differences in beam arrangement and natural heart and lung shielding may play a role. Though this trend was not statistically significant, it aligns with literature suggesting laterality can influence organ-at-risk dose exposure and, by extension, toxicity outcomes.

Moreover, our findings on mean lung dose (MLD) are similar to those reported by Salim et al. [38]. These early radiological changes indicate subclinical manifestations of radiation-induced lung injury and may precede later fibrotic remodeling, which is especially pertinent for long-term breast cancer survivors. These findings suggest that the development of radiological injury in this cohort was not linked to higher lung dose exposure.

However, based on our findings, mastectomy status alone does not appear to be a decisive factor in RILI risk, although its interaction with specific radiotherapy parameters warrants further exploration.

A central question in this study was whether COVID-19 vaccination, through potential modulation of immune responses, might influence the development of post-radiotherapy RILI. Our results, derived from multiple statistical methods including Chi-square test (*p* = 0.3569) and logistic regression analysis (*p* = 0.646, OR = 1.063). The lack of statistical significance in our analysis does not definitively prove that no association exists. Rather, our findings should be interpreted as insufficient evidence to confirm or exclude a relationship between COVID-19 vaccination and radiation-induced lung injury, due to the limited sample size and retrospective design. The very low pseudo R² value of the regression model (0.00098) further emphasizes the lack of explanatory power. This is consistent with prior studies suggesting that COVID-19 vaccines, although capable of transiently activating immune responses, do not substantially alter chronic inflammatory pathways involved in radiation-induced lung injury [9, 10, 15, 39]. From a biological perspective, COVID-19 vaccination may influence pulmonary responses through immune modulation or inflammatory pathways. Vaccination induces transient immune activation, raising theoretical concerns that this could exacerbate radiation-induced lung toxicity. However, recent clinical and experimental studies suggest that COVID-19 vaccines do not lead to long-term pulmonary inflammation, and large-scale population data have not shown persistent fibrotic changes attributable to vaccination [40, 41, 42, 43]. Our findings align with these reports, indicating that prior vaccination does not increase the risk of radiation-induced lung injury in breast cancer patients.

Finally, the distribution of vaccine doses and RILI status did not reveal any meaningful pattern, despite a slightly lower RILI rate among patients who received exactly two doses (22 vs. 9). Taken together, our results suggest that while several patient-related and treatment-related factors may influence the risk of post-radiotherapy pulmonary injury, none of the examined variables—including COVID-19 vaccination status, age, lung volume, treatment laterality, or mastectomy—emerged as strong, independent predictors in our cohort. This highlights the multifactorial nature of radiation-induced lung injury and underscores the importance of comprehensive dosimetric analysis and individualized treatment planning. Future studies incorporating detailed dose-volume histogram data and molecular or genetic susceptibility markers could provide deeper insights into the mechanisms underlying RILI risk and help identify patients at higher risk.

The following limitations must be noted:

It is essential to acknowledge the limitations of this study. The retrospective and single-center design restricts the generalizability of the findings. Additionally, detailed information on important potential confounders was not consistently available in the institutional registry, including prior chemotherapy, hormone therapy, smoking history, and pre-existing pulmonary comorbidities (e.g., COPD, interstitial lung disease). As a result, these data could not be included in the analysis. These factors may not have been uniformly distributed across the various vaccination groups and could have influenced the observed outcomes.

Furthermore, the modest sample size reduced statistical power and precluded definitive conclusions regarding subtle associations between vaccination and RILI risk. The variability in follow-up imaging schedules and the absence of histopathological confirmation of RILI may have introduced further bias. Collectively, these limitations highlight the need for larger, multicenter, prospective studies with comprehensive clinical data. While all vaccinated patients received their doses before the initiation of radiotherapy, information on booster doses administered afterward was incomplete. Additionally, the reasons for non-vaccination could not be fully established, although they likely relate to age or contraindications. Information on vaccine type (mRNA vs. vector-based) was not consistently available in the medical records and, therefore, could not be analyzed in this study.

We must note that the combined use of chest CT and X-ray images may have led to an underestimation of grade 1 subtle changes. However, this potential limitation is unlikely to be systematically related to COVID-19 vaccination status.

Although COVID-19 vaccination status prior to radiotherapy was included in the present analysis, information on booster vaccinations administered after completion of radiotherapy was incomplete and therefore not systematically analyzed. Post-radiotherapy immune activation related to subsequent booster vaccinations could theoretically influence radiation-induced lung injury or late fibrotic changes, particularly in previously irradiated lung tissue. As such, post-RT booster vaccination should be considered a potential confounding factor when interpreting the present findings. Future prospective studies with comprehensive longitudinal vaccination data are warranted to further clarify the interaction between post-radiotherapy immune stimulation and radiation-induced pulmonary toxicity.

## Conclusions

Our results show that COVID vaccination prior to radiation therapy is unlikely to negatively affect pulmonary toxicity outcomes. Although a slight protective effect was observed with two vaccine doses, it was not statistically significant.

Due to the retrospective nature of the study and the limited sample size, larger, prospective, long-term follow-up studies are needed to understand the deeper immunological effects.

Future research should also examine whether certain patient groups, particularly those with pre-existing lung disease, respond differently to vaccination in the context of radiation-induced lung damage. A deeper understanding of the effects of COVID-19 vaccination on lung regeneration and the immune response after radiation therapy may improve oncology treatment strategies.

## Data Availability

The datasets generated and/or analyzed during the current study are available from the corresponding author upon reasonable request.
